# Sertraline, citalopram and paroxetine in lactation: passage into breastmilk and infant exposure

**DOI:** 10.3389/fphar.2024.1414677

**Published:** 2024-05-22

**Authors:** Daphne Den Besten-Bertholee, Daan J. Touw, Elvera A. Damer, Paola Mian, Peter G. J. Ter Horst

**Affiliations:** ^1^ Department of Clinical Pharmacy, Isala, Zwolle, Netherlands; ^2^ Groningen Research Institute of Pharmacy, Section Pharmaceutical Analysis, University of Groningen, Groningen, Netherlands; ^3^ Department of Clinical Pharmacy and Pharmacology, University of Groningen, University Medical Centre Groningen, Groningen, Netherlands; ^4^ Department of Psychiatry, Isala, Zwolle, Netherlands

**Keywords:** breastfeeding, antidepressants, selective serotonin reuptake inhibitors (SSRIs), infant dose, lactation

## Abstract

**Objectives:**

This study aimed to investigate the plasma and breastmilk concentrations for sertraline, citalopram and paroxetine for assessment of the Milk/Plasma (M/P) ratio and Absolute Infant Dose (AID), and to determine actual infant drug exposure through breastfeeding. Subsequently, informed recommendations will be formulated regarding the advisability of breastfeeding in women undergoing treatment with the three most widely used antidepressants.

**Methods:**

A pharmacokinetic study in lactating women and their infants using sertraline, citalopram or paroxetine was performed. Paired breastmilk and plasma samples and single point infant plasma samples were collected to determine antidepressant concentrations. An Area Under the Curve (AUC) based approach with the trapezoidal rule was used to calculate M/P ratios and AID for all three antidepressants by combining all measured concentrations for the same dose.

**Results:**

Thirty-seven lactating women and their infants participated in this study. 111 paired breastmilk and plasma samples and 37 single point infant plasma samples were collected. Detectable concentrations of sertraline, citalopram and paroxetine were present in all breastmilk samples. For sertraline and citalopram M/P ratio is above one, indicating higher breastmilk than plasma concentrations, however, drug exposure by breastmilk did not lead to detectable plasma drug levels in any of the 15 infants for sertraline, for nine (out of 13) infants for citalopram and for eight (out of nine) infants for paroxetine.

**Conclusion:**

Given the well-known benefits of breastfeeding, our findings support breastfeeding of infants by mothers who are taking sertraline, citalopram or paroxetine is safe. Sertraline and paroxetine are the preferred antidepressants during breastfeeding, reaching mostly undetectable infant drug levels.

## Introduction

The number of mothers who breastfeed their infant has increased up to 97%, with rates differing largely between countries (Theurich et al., 2019; Centers for Disease Control and Prevention, 2023). Human milk provides essential nutrients for the infant and provides health benefits for both mother and child. Breastmilk increases the mother-child bonding, strengthens the immune system and stimulates the gastrointestinal function of the infant (Kakuma, 2012). Because of these health benefits and the low costs of breastfeeding, the World Health Organization recommends exclusive breastfeeding during the first 6 months of life and up to 2 years as supplement (World Health Organization, 2024).

The prevalence of depression is 10%–15% in pregnant women (Evans et al., 2001; Nilam, 2015), and 5%–29% in the *postpartum* period (Gaffney et al., 2014; Howell et al., 2014; Shu-Yu et al., 2014). 2.9%–5.5% of nursing mothers use antidepressants (Tessa et al., 2006; Margulis Andrea et al., 2014). Reported side effects in the neonate due to possible exposure of antidepressant medication through breastmilk are somnolence, lethargy, fever, reduced postnatal growth, and excessive infant crying (Hale et al., 2010; Uguz and Arpaci, 2016). On the contrary, discontinuing antidepressants during breastfeeding, which seems to overcome adverse drug reactions in the suckling infant, may lead to bonding problems, adverse child development, excessive infant crying, and a possible relapse of maternal depression (Wai and Green, 2009; Cristie et al., 2010). Selective Serotonin Reuptake Inhibitors (SSRIs) are the most used antidepressants during pregnancy and lactation, because tricyclic antidepressants are not recommended in this population (Andrade et al., 2008; Molenaar et al., 2019). Investigating the safety of antidepressant use while breastfeeding is difficult because of ethical and logistic issues. The Food and Drug Administration (FDA) and European Medicines Agency (EMA) guidelines state that the Absolute Infant Dose (AID) is one of the major elements for a risk assessment of using antidepressants during breastfeeding (Food and Drug Administration and FDA, 2005; European Medicines Agency, 2008).

Furthermore, when the AID is known, the Relative Infant Dose (RID) can be determined. The relative infant dose provides a relative measure of the infant’s exposure compared to the maternal dose, helping healthcare professionals determine the potential risk to the nursing infant. In literature, a RID of less than 10% is generally considered safe (Begg et al., 2002).

The EMA guideline also classified infant drug plasma concentrations as a major element of assessment, since this is the only direct parameter of infant exposure (European Medicines Agency, 2008).

Quantitative studies assessing the AID or Milk/Plasma ratio (M/P ratio) correctly, are of clinical importance to relate possible side effects in the suckling infant to the concentration of antidepressants in breastmilk. A review of literature showed that most lactation studies with antidepressants did not provide an AID or M/P assessed according to FDA guidelines and described earlier by Begg et al., 2002 (den Besten-Bertholee et al., 2019).

A prerequisite for counselling breastfeeding mothers using antidepressants is that data on M/P ratio and AID are available, to comprehensively assess the potential risks posed by antidepressant exposure to the nursing neonate. Therefore the aim of this study was to investigate the plasma and breastmilk concentrations for the three most widely used antidepressants (citalopram, paroxetine and sertraline) for assessment of the M/P ratio and AID according to the relevant guidelines, and to determine actual infant drug exposure through breastfeeding. Subsequently, informed recommendations will be formulated regarding the advisability of breastfeeding in women undergoing treatment with sertraline, citalopram, or paroxetine.

## Materials and methods

### Patients

This pharmacokinetic study was conducted at the Isala teaching hospital in Zwolle, the Netherlands. The local research ethics committee reviewed the protocol and concluded there was minimal patient burden of the additional Dried Blood Spot (DBS) and breastmilk sampling (reference number 14.0677). Patients approved participation in the study by informed written consent prior to enrollment. Inclusion criteria were lactating women (>4 weeks *postpartum*) on steady state (>8 days current dose) of sertraline, citalopram or paroxetine, with adequate understanding of the Dutch language. From February 2014-February 2018 all eligible women giving birth at our hospital were asked to participate in this study.

### Sample collection

To minimize patient burden DBS sampling was used by finger prick technique using a limited sampling strategy according to (Ilett et al., 2008). The sample collection times for patients were determined according to a predetermined schedule over one dose interval (Supplemental Information). Participating women were asked to collect DBS and breastmilk samples simultaneously at home, on three time points in 2 days. They were instructed to record the exact times of drug intake and sampling. Patients received detailed instructions according to Clinical & Laboratory Standards Institute (CLSI) guidelines for correct DBS sampling (Protein Saver™ 903™ Card, Whatman™ GE Healthcare Bio-Sciences Corp Westborough, United States) using a microtainer lancet. On the third day a DBS sample was collected from the infant by heel puncture, performed at our hospital by trained nurses.

Breastmilk samples were collected by fully emptying one breast using an electric breast pump. The sample was gently mixed and 5 mL was collected and stored in the patient’s home freezer at −18°C. Remaining breastmilk was fed to the suckling infant. All samples were handed over at day 3 when the infant sample was collected at the hospital. All breastmilk samples were stored in de laboratory at −80°C and DBS samples were stored −20°C until analysis.

### Analytical methods

Sertraline, citalopram and paroxetine concentrations in DBS and breastmilk were determined with an Ultra Performance Liquid Chromatography tandem Mass Spectrometry (UPLC-MS/MS) system.

A 10-mm diameter sample was punched out of the DBS card and 200 µL extraction solution with internal standard was added. We used 20 ng/mL sertraline-d3 (Cerilliant, Round Rock, Texas, United States) 0.1 μg/mL citalopram-d6 (Cerilliant, Round Rock, Texas, United States) and 1 μg/mL paroxetine-d4 (Toronto Research Chemicals Inc, North York, Canada) solutions as internal standards in acetonitrile/methanol (2:1). The samples were analysed using an in house developed analytically and clinically validated UPLC-MSMS method (Waters Acquity™ C18, 1.7 um; 2.1 × 50 mm column and VanGuard 2.1 × 5 mm pre-column at 50°C, TQD detector Electrospray + mode).

The assay demonstrated linearity in the range 2–224 μg/L (sertraline), 4–294 μg/L (citalopram), 6–150 μg/L (paroxetine). Breastmilk samples (50 µL) were analyzed using the same technique, with calibration curves from medication-free voluntary donated human breastmilk. Inter- and intra assay variations were all below 5%. Passing Bablok regression analysis of the eight-point calibration curves showed no matrix effects.

### Clinical validation

To define the differences between standard EDTA plasma concentrations and concentrations from capillary blood obtained by fingerprick and applied on a DBS card, a clinical validation study was performed. This study is described in detail in de online supplementary material. A correction factor was used to convert DBS concentrations to plasma concentrations for sertraline and citalopram. The equations used are:

$$\left[plasma\;sertraline\right]=0.656*\left[DBS\;sertraline\right]$$

$$\left[plasma\;citalopram\right]=0.562*\left[DBS\;citalopram\right]$$

For paroxetine no conversion factor was needed.

### Statistical analysis

Area under the curve (AUC) for breastmilk and plasma was calculated using the trapezoidal rule for every antidepressant by combining all measured concentrations for the same dose. M/P ratio was calculated by dividing the *AUC*_*breastmilk*_ *with the AUC*_*plasma.*_ The AID (mg/kg/day) was calculated by multiplying the AUC of breastmilk concentrations with the daily intake of 0.15 L breast milk per kg per day, which is the average milk intake value of a 2-month old infant, who is solely breastfed (Begg et al., 2002). Concentrations of the antidepressant at less than the lower limit of quantification (LLOQ) were assessed as half of the lower limit.

All statistics and calculations were performed using Rstudio^®^ software (Version 4.2.1).

## Results

Thirty-seven lactating women and their infants participated in this study. Sertraline was the most used drug (*n* = 15), followed by citalopram (*n* = 13) and paroxetine (*n* = 9). 111 paired breastmilk and plasma samples and 37 single point infant plasma samples were collected. Demographic characteristics, including data on daily doses, weight and age are shown in Table 1. In total, three DBS samples where discarded because of insufficient quality, all from the same patient using paroxetine. One citalopram breastmilk sample was not collected by the subject.

**TABLE 1:** Summary of demographic characteristics. Values are shown as mean (range).

	Sertraline	Citalopram	Paroxetine
Mothers/Infants, n	15/15	13/13	9/9
Maternal drug dose, mg/d	83 (50–200)	21 (10–40)	29 (5–40)
Weight mother, kg	79 (61–110)	79 (57–105)	75 (63–95)
Weight infant, kg	3.8 (2.7–5.8)	4.3 (2.7–5.7)	4.8 (3.2–8.0)
Age infant, months	1.4 (1.0–2.6)	1.5 (0.7–3.3)	1.7 (1.5–9.0)

Table 2 outlines the mean plasma and breastmilk concentration, AUC, M/P ratio, AID, RID and single point infant drug concentration for all three drugs. All concentrations were within the range of our assay.

**TABLE 2:** Mean plasma and breastmilk concentration (range), AUC (range), M/P ratio, AID, RID and single point infant drug concentration (range) for all three drugs.

	Sertraline	Citalopram	Paroxetine
Plasma concentration, µg/L	41 (5–200)	63 (16–131)	130 (34–223)
Milk concentration, µg/L	87 (12–288)	119 (36–274)	63 (<6–142)
AUC_plasma_	725 (417–1185)	1432 (417–2814)	1473 (1055–1857)
AUC_milk_	1670 (1029–2219)	2578 (902–4139)	859 (600–1111)
M/P ratio	2.3	1.8	0.6
AID, µg/kg/dag	10	16	5
RID,%	1	6	2
Single point infant drug concentration, µg/L	<2 (<2–2.6)	4 (<4–15)	<6 (<6–6.4)

Detectable concentrations of sertraline (12–288 μg/L), citalopram (36–274 μg/L) and paroxetine (1–42 μg/L) were present in all breastmilk samples. The drug exposure by breastmilk did not lead to detectable plasma drug levels in any of the 15 infants for sertraline, for nine (out of 13) infants for citalopram and for eight (out of nine) infants for paroxetine. For sertraline and citalopram M/P ratio is above one, indicating higher breastmilk than plasma concentrations.

**FIGURE 1:**
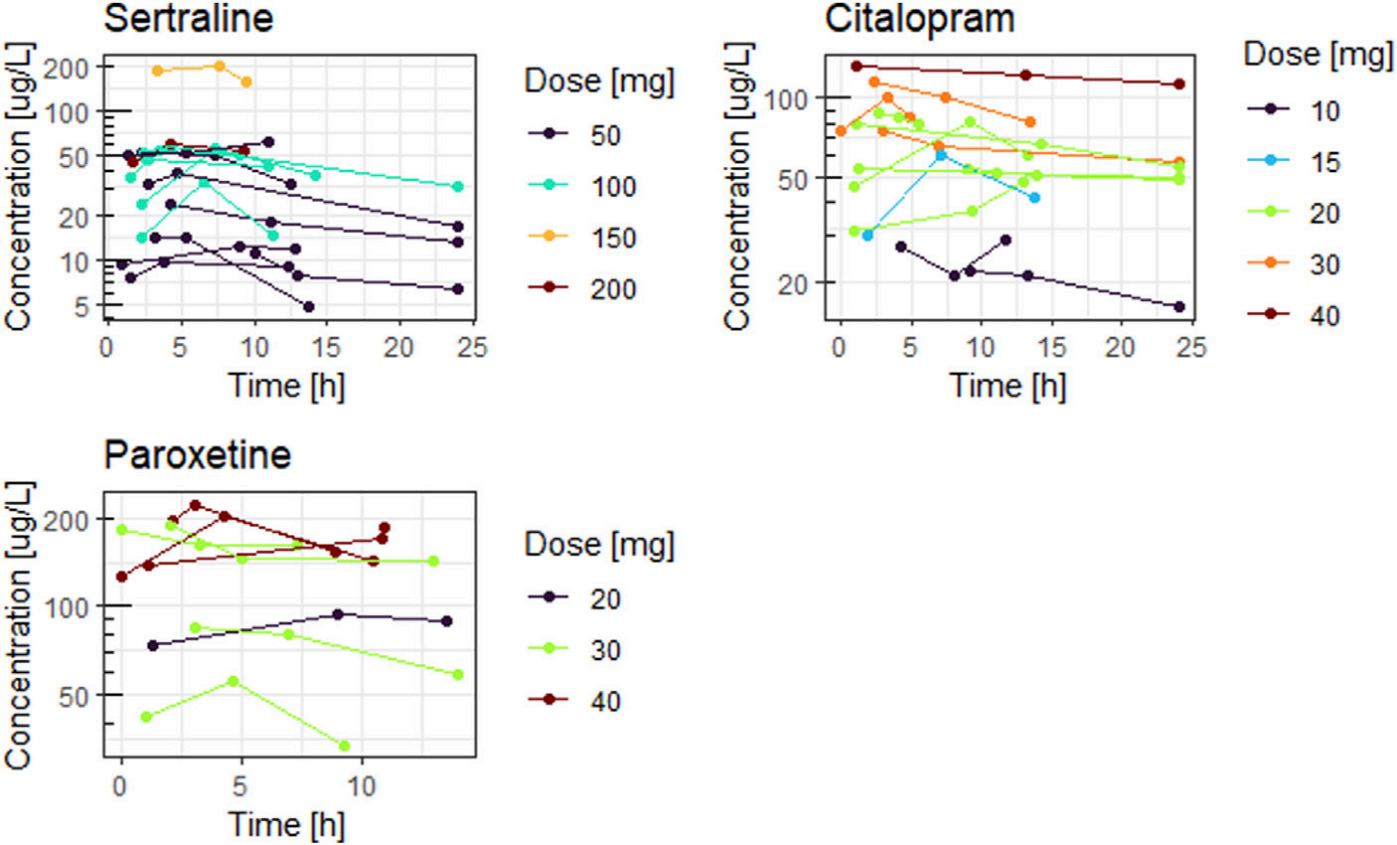
Concentration time curves in plasma by dose.

**FIGURE 2:**
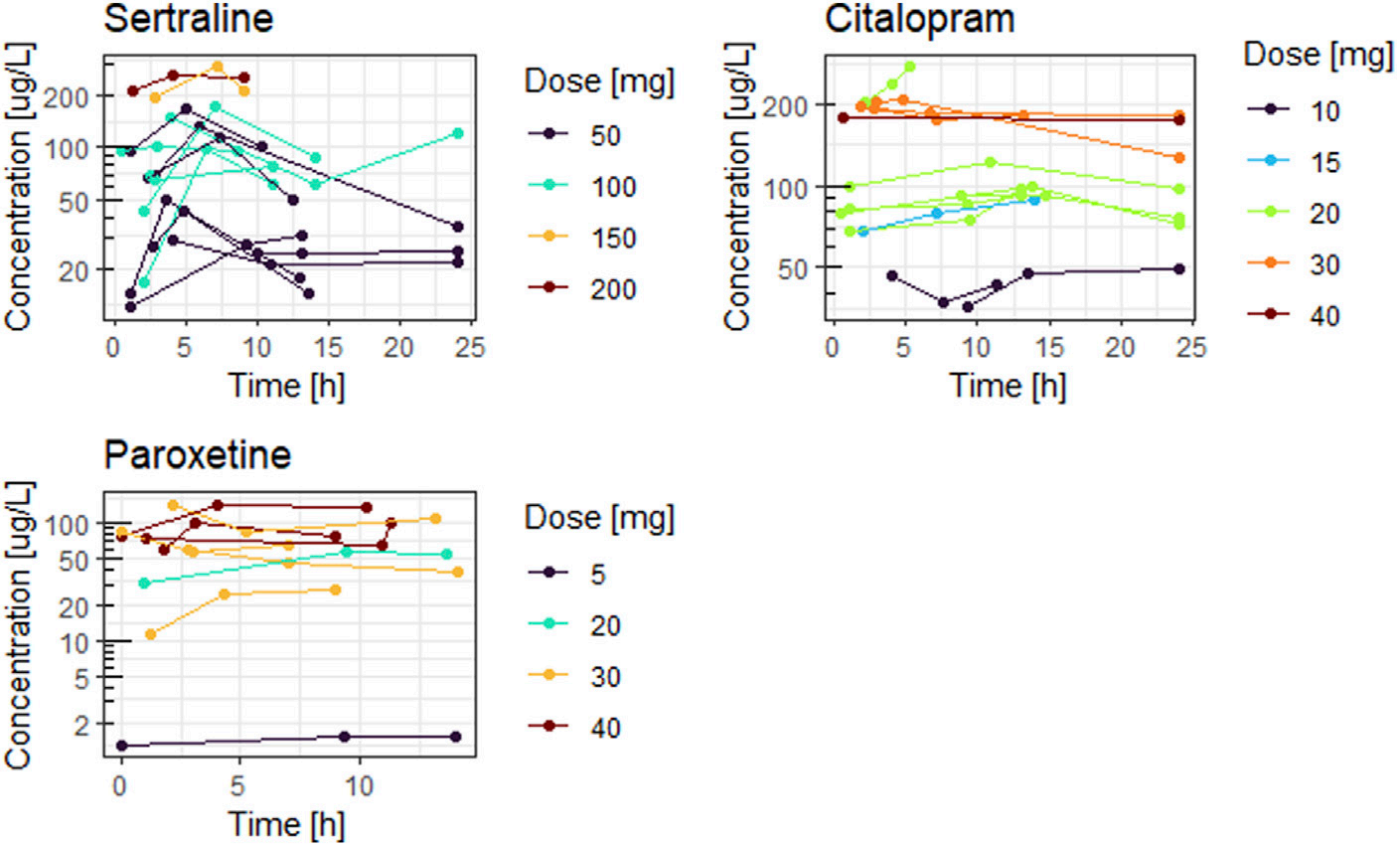
Concentration time curves in breastmilk by dose.

Figures 1, 2 show the concentration time curves in plasma and breastmilk. For all three antidepressants, the concentrations in both breastmilk and plasma increase with increasing dose, although there is no linear correlation. For sertraline, concentrations in breastmilk tend to decline over time after dose. For citalopram and paroxetine no evident decline was observed.

**FIGURE 3:**
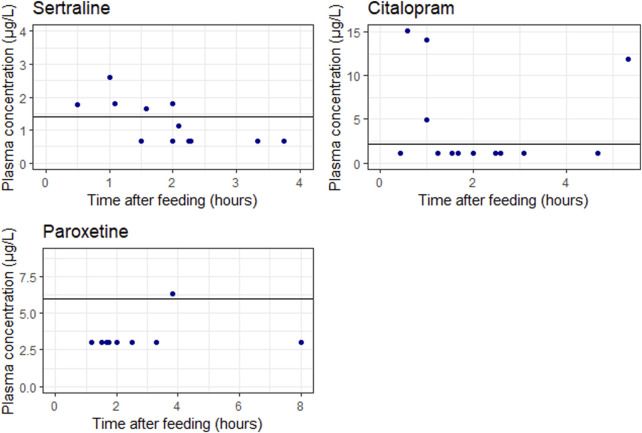
Measured concentrations in neonates related to the time after last breastfeeding. The blue points represent the individual measurements. The solid lines represents the lower limit of quantification (LLOQ). It should be noted that one sertraline concentration is higher than half the LLOQ but below the LLOQ line, this is explained because the actually measured DBS concentration was higher than the LLOQ.

Figure 3 shows measured concentrations in neonates related to the time after last breastfeeding. No evident correlation between concentrations measured and time after last feeding is observed. Furthermore, a majority of the concentrations fell below the lower limit of quantification.

## Discussion

The present study adds quality data on the M/P ratio, AID and infant exposure for sertraline, citalopram and paroxetine in a relatively large group of breastfeeding mothers and their infants.

As described earlier, data on the extent of antidepressant drug transfer into breastmilk and subsequent exposure to the suckling infant is sparse and do not always meet the FDA and EMA guidelines (den Besten-Bertholee et al., 2019).

Our results show that all three antidepressants are excreted in breastmilk, with for sertraline and citalopram an M/P ratio above one, indicating higher breastmilk than plasma concentrations. This is possibly because of the high lipophilicity of the drugs. For sertraline an AUC based M/P ratio of 2.3 was calculated, which is comparable to earlier reported values (Kristensen et al., 1998; Dodd et al., 2000; Stowe et al., 2003), although with high inter-individual variability. For citalopram the calculated M/P ratio was 1.8, which is also in line with earlier reported values (Schoretsanitis et al., 2019). For paroxetine, the concentrations were lower in breastmilk than in plasma, with an M/P ratio of 0.6. Pogliani et al., 2019 reported M/P ratio’s for paroxetine of 0.6–1.2, although no AUC based approach was used.

Overall it has to be noted that M/P ratio is only an indicator of drug transfer properties and does have little value in predicting infant exposure (Ilett and Kristensen, 2005). Therefore, another important parameter in the risk assessment according to the FDA is the absolute infant dose (AID). An AID for sertraline of 10 μg/kg/day for citalopram 16 μg/kg/day and for paroxetine 5 μg/kg/day was found. Schoretsanitis *et al.* found a similar AID for citalopram with 17 μg/kg/day and a lower AID for sertraline with 2 μg/kg/day (Schoretsanitis et al., 2019) Dodd *et al.* found a similar median AID of 7 μg/kg/day for sertraline (Dodd et al., 2000). In both studies, no AUC based approach was used. Furthermore, when the AID is known, the relative infant dose (RID) can be determined. In our study, the RID was less than 10% for sertraline (1%), citalopram (6%) and paroxetine (2%), which is generally considered safe in literature (Begg et al., 2002). However, when the mother is using a high dose of the antidepressant, a RID of less than 10% can still lead to a high infant exposure. Therefore, the actual measured infant concentrations are a relevant parameter in the risk assessment.

For sertraline and paroxetine only low and often undetectable concentrations were measured in the breast fed infant, despite high concentrations in breastmilk. For citalopram however, detectable concentrations (up to 15 μg/L) are measured in the breastfed infant when the citalopram dose is ≥ 20 mg/day.

Results show sertraline intake through breastfeeding does not lead to detectable infant plasma concentrations. We hypothesize this is because of different pharmacokinetics in neonates, such as absorption and volume of distribution (Kearns et al., 2003). To test this hypothesis we collected a feces sample of one breastfed infant and measured a high sertraline concentration of 501 μg/L and desmethylsertraline of 2874 μg/L. Neonates physiology differs substantially compared with older children and adults, including enzyme expression and maturation, affecting drug disposition. It is described this leads to unexpectedly low bioavailability of weakly acidic drugs (such as sertraline) (Zimmerman et al., 2019). Further research is needed to confirm our hypothesis.

One of the major strengths is the AUC based approach of our study, with multiple measurements at different times of the day throughout a large part of the dosage interval, since breastmilk composition such as lipid content fluctuates over the course of a day (Khan et al., 2013). The design of the study included home sampling and therefore had limited interference in the daily life for the breastfeeding mother and her infant.

It should be noted that because we used DBS techniques to minimize patient burden, we actually measured whole blood levels instead of plasma levels. A correction factor was used to convert DBS concentrations to plasma concentrations for sertraline and citalopram. Although the correlation was high, it is possible it introduces a small error. However, measured concentrations and M/P ratios are similar to those described earlier in literature supporting the validity of the correction factors used.

A limitation of our study is we did not measure concentrations of major metabolites desmethylsertraline and desmethylcitalopram. However, the activity of these metabolites is low compared with the parent compound and therefore the contribution to the efficacy or tolerability is negligible (Hiemke et al., 2018). Genetic polymorphisms, specifically CYP2D6 and CYP2C19, have been shown to impact plasma concentrations of sertraline, citalopram and/or paroxetine (Hiemke et al., 2018). Our study did not consider these variations, although it could have explained some of the inter-individual differences in plasma concentrations seen within the same dose.

Our data strengthen the current advice on LactMed^®^ that most authoritative reviewers consider sertraline and paroxetine the preferred antidepressants during breastfeeding (Drugs and Lactation Database, 2006). However, discontinuing effective antidepressant treatment in the *postpartum* period should be avoided, and switching from another antidepressant to sertraline or paroxetine might be problematic in this vulnerable period (Berle and Spigset, 2011). When citalopram is indicated there is no reason not to recommend breastfeeding because of minimal infant exposure in relation to all described breastfeeding health benefits (Kakuma, 2012).

In conclusion, given the well-known benefits of breastfeeding, these findings support breastfeeding of infants by mothers who are taking sertraline, citalopram or paroxetine is safe.

## Data Availability

The raw data supporting the conclusion of this article will be made available by the authors, without undue reservation.

## Abbreviations

SSRI, Selective serotonin reuptake inhibitors; FDA, Food and Drug Administration; EMA, European Medicines Agency; AID, Absolute Infant Dose; RID, Relative Infant Dose; M/P, Milk/Plasma; DBS, Dried Blood Spot; AUC, Area Under the Curve; LLOQ, Lower Limit Of Quantification.
