# Supplementary material for: Sertraline, citalopram and paroxetine in lactation: passage into breastmilk and infant exposure
Source: Front Pharmacol. 2024 May 22;15:1414677. doi: 10.3389/fphar.2024.1414677 (PMC11150716; doi:10.3389/fphar.2024.1414677)
Supplement: Supplementary file 1 [file Table1.DOCX]

Supplemental Data

The sample collection times for patients were determined according to a predetermined schedule over one-dose interval.
*t: time after dose intake (hours)
P: patient
*Immediately before intake morning dose
Green = day 1 Red with diagonal line = day 2*

| **t**  **P** | **0*** | **1** | **2** | **3** | **4** | **5** | **7** | **9** | **11** | **13** | **14** |
| --- | --- | --- | --- | --- | --- | --- | --- | --- | --- | --- | --- |
| **1** |  |  |  |  |  |  |  |  |  |  |  |
| **2** |  |  |  |  |  |  |  |  |  |  |  |
| **3** |  |  |  |  |  |  |  |  |  |  |  |
| **4** |  |  |  |  |  |  |  |  |  |  |  |
| **5** |  |  |  |  |  |  |  |  |  |  |  |
| **6** |  |  |  |  |  |  |  |  |  |  |  |
| **7** |  |  |  |  |  |  |  |  |  |  |  |
| **8** |  |  |  |  |  |  |  |  |  |  |  |
| **9** |  |  |  |  |  |  |  |  |  |  |  |
| **10** |  |  |  |  |  |  |  |  |  |  |  |
| **11** |  |  |  |  |  |  |  |  |  |  |  |
| **12** |  |  |  |  |  |  |  |  |  |  |  |
| **13** |  |  |  |  |  |  |  |  |  |  |  |
| **14** |  |  |  |  |  |  |  |  |  |  |  |
| **15** |  |  |  |  |  |  |  |  |  |  |  |
