# Supplementary material for: Sertraline, citalopram and paroxetine in lactation: passage into breastmilk and infant exposure
Source: Front Pharmacol. 2024 May 22;15:1414677. doi: 10.3389/fphar.2024.1414677 (PMC11150716; doi:10.3389/fphar.2024.1414677)
Supplement: Supplementary file 2 [file DataSheet1.PDF]

Analytical and clinical validation of an Liquid Chromatography-Mass Spectrometry method for sertraline, citalopram and paroxetine in Dried Blood Spots

This study aims to analytically validate a method to determine the concentrations of sertraline, citalopram and paroxetine in DBS and to investigate the correlation between citalopram, sertraline and paroxetine drug concentrations in venous blood and DBS samples in the same patients drawn at the same time.

Materials and methods

Patients and sample collection

Patient samples were collected at the Isala teaching hospital Zwolle, the Netherlands. A waiver was received from the local research ethics committee because of minimal patient burden of the additional DBS sampling sampling. Patients approved participation in the study by signing informed consent prior to blood sampling. Inclusion criteria were adult patients on routine therapeutic drug monitoring (TDM) for citalopram, sertraline or paroxetine with adequate understanding of the Dutch language. From April 2015 until February 2019 all eligible patients, men and women, were asked to participate in this study. Venous samples for routine TDM were collected by trained laboratory staff in a 5 ml EDTA collecting tube. All samples reflected trough drug concentrations. DBS samples by finger puncture were collected at the same time as routine TDM samples for plasma and did not require extra visits to the clinic. Blood (five drops) was collected by the patients on DBS filter paper (Protein Saver™ 903™ Card, Whatman™ GE Healthcare Bio-Sciences Corp Westborough, USA) using a microtainer lancet. Guidelines from the Clinical & Laboratory Standards Institute (CLSI) were followed. Patients were instructed to warm their hands before the fingerprick and to wipe away the first drop of blood. DBS cards were dried to air at room temperature for 4 hours before storage in ziplock biohazard bags at -20°C until they were analyzed.

Analytical method

Citalopram, sertraline and paroxetine were measured in both plasma and DBS using an in- house developed and validated a Liquid Chromatography-Mass Spectrometry (LC-MS-MS) method according to Guidance for Industry: Bioanalytical Method Validation, by the U.S. Food and Drug Administration.
The assay was performed with Waters Acquity Ultra Performance (Milford, MA, USA) coupled with a Waters Acquity Xevo triple quadrupole mass spectrometer (TQD) (Milford, MA, USA). The used column is a Waters Acquity UPLC BEH Phenyl 1.7 μm; 2.1x50 mm column and the column temperature was set at 50 °C. The eluents used are A: formic acid (0.1%) in water and B: formic acid (0.1%) in acetonitrile. The flow rate of the mobile phase was 0.6 mL/min. For sertraline, mobile phase gradient started with 65%(A):35%(B) at 0 min, 55%(A):45%(B) between 1.0-1.1 min, to 0%(A):100%(B) between 1.1-1.5 min and 65%(A):35%(B) from 1.5 min. The total runtime was 1.8 min. For citalopram and paroxetine, mobile phase gradient started with 75%(A):25%(B) at 0 min, 65%(A):35%(B) between 4.5-5.0 min, to 0%(A):100%(B) between 5.0-5.5 min and 75%(A):25%(B) from 5.5 min. The total runtime was 5.8 min. The autosampler temperature was set at 10 °C. The volume of injection was a 20μL full loop. The ionisation mode is Electrospray positive (ES+). The source temperature was set at 140 °C and the desolvation temperature was set at 400 °C. The cone gas (nitrogen) flow was set to 50 L/h, the desolvation gas (nitrogen) flow was set to 800-1000 L/h and the collision gas (argon) flow was set to 0.20 mL/min. The voltage of the capillary was 0.50-0.80 kV, of the extractor 3.00 V and the Rf was 0.20 V. We used Masslynx software to calculate concentrations. All m/z values for transitions, cone and collision energy values were tuned and optimized in our laboratory and are shown in Table 1.

Table 1 Mass spectrometer settings for all substances

Component	m/z transition	Cone (V)	Collision energy (V)
Sertraline	306.00 > 159.03	8	20
Sertraline-D3	309.00 > 159.03	8	20
Citalopram	325.20 > 109.00	40	25
Citalopram-D6	331.25 > 109.00	40	25
Paroxetine	330.20 > 69.70	38	28
Paroxetine-D4	334.20 > 73.70	38	28

m/z, mass-to-charge ratio

Sample preparation and calibration standards

A sample with a 10 mm diameter was punched out of the DBS card. The sample was transferred to a test tube, soda glass round bottom provided by VWR International Europe BVBA (Leuven, Belgium), and 200 µL of extracting solution was added. The extracting solution consisted of 20 ng/mL sertraline-D3 (Cerilliant, Round Rock, Texas, USA), 0.1 mg/L citalopram-D6 (Cerilliant, Round Rock, Texas, USA) and 1.0 mg/L paroxetine-D4 (Toronto Research Chemicals, North York, Canada) as internal standards in methanol/acetonitrile (2:1). After 10 minutes of vortex, 75 µL of the extract was transferred to a 2 mL Square Collection Plate, further 75 µL milli-q water (Millipore, Darmstadt, Germany) for sertraline and 300 µL milli-q water for citalopram/paroxetine was added to the vials and 20 µL was injected into the LC-MS. For every patient, we analysed two different blood spots, with an acceptance limit of <15% between both results.

The calibration standards and independent quality control standards were prepared by spotting 65 µL of spiked blood (HCT 0.38-0.42 v/v) onto the DBS cards, extracted and quantified the same way as patient samples.

Influence of spot volume and haematocrit

The impact of different spotted blood volumes (20, 40, 65, 80 and 100 µL) at two concentration levels of sertraline (12 and 150 µg/L), citalopram (12 and 150 µg/L) and paroxetine (6 and 75 µg/L) was tested. The impact of haematocrit was evaluated by preparing blood with different haematocrit ranges (0.4–0.8) and spiking 65 µL with the abovementioned two concentration levels of sertraline, citalopram and paroxetine.

Clinical validation

Concentrations measured in plasma and DBS were compared using Passing Bablok regression analysis using Rstudio® software (Version 4.2.1). Passing Bablok regression analysis tests for a constant bias and proportional bias between two different validated methods and allows for measurement errors on both the x and y-axis and is not influenced by a few outliers. If the 95% confidence interval (CI) for the intercept of the regression line includes 0, no significant differences between the methods are observed. If the 95% confidence interval for the slope of the regression line includes 1, there is no proportional bias between the two methods tested.

Results

Patients

A total of 47 paired patient samples were included in this clinical validation study; 14 citalopram, 29 sertraline and 4 paroxetine samples. All concentrations fell within the validated range, with the exception of one high citalopram DBS concentration. This sample was diluted and subsequently reanalyzed.

Analytical validation

The assays were validated for both DBS and plasma over the ranges 2-224 µg/L for sertraline, 4-294 µg/L for citalopram and 6-150 µg/L for paroxetine with correlation coefficients of respectively 0.9998, 0.9991 and 0.9996. Within-day and between-day precision showed highest RSD 4.9% (DBS) and 2.1% (plasma) for citalopram, 4.6% (DBS) and 2.9% (plasma) for sertraline and 4.5% (DBS) and 6.0% (plasma) for paroxetine. Maximum bias was for citalopram +6.6% (DBS) and -6.8% (plasma), for

sertraline +5.3% (DBS) and +10.4% (plasma) and for paroxetine +3.3% (DBS) and -13.0% (plasma). Both precision and accuracy thereby met pre-set acceptance criteria (≤ 15%). No carry-over was observed and all results for recovery and matrix effects were within 85-115% range.
Validation samples were observed to be stable for at least six months when stored in ziplock biohazard bags at -20 degrees (RSD ≤ 15% related to the nominal concentration at 3 different QC levels).

Influence of spot volume and haematocrit
For all three antidepressants, only for a spotted volume of 20 µL a bias of >15% was observed. The impact of haemotocrit was maximally 17%.

Clinical validation
Figure 1 shows the Passing Bablok regression analysis plotting the plasma versus DBS concentrations. For all three antidepressants, plasma and DBS concentrations correlated highly (R^2=0.99 for sertraline, R^2=0.95 for citalopram and R^2=0.98 for paroxetine), indicating a linear relationship. For sertraline and citalopram, concentrations were lower in plasma samples compared to DBS samples (slope 0.66 and 0.56 respectively), meaning a conversion factor is needed. Concentrations measured in DBS can be converted to corresponding plasma concentrations using the following equations:

$$[plasma\ sertraline] = 0.656 * [DBS\ sertraline]$$
$$[plasma\ citalopram] = 0.562 * [DBS\ citalopram]$$

For paroxetine no differences were observed, implying no conversion factor is needed.

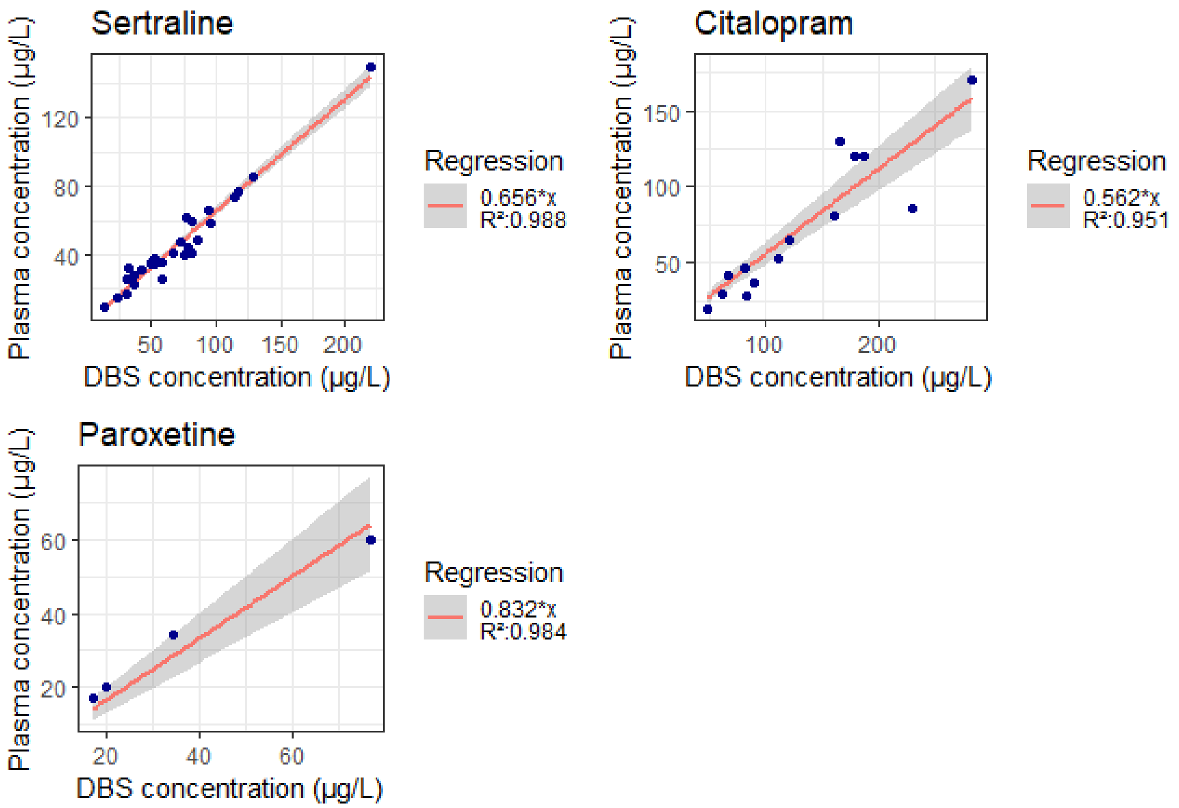

Figure 1. Passing-Bablok regression analysis plotting plasma concentrations against dried blood spots (DBS) concentrations. The blue points represent the individual measurements. Regression line (orange) with 95% confidence intervals (grey area).

Conclusion
The method was successfully validated for citalopram, sertraline and paroxetine and will be used for determination of the Milk/Plasma ratio in our lactation studies, applying a conversion factor for sertraline and citalopram.
